# Interventions promoting mental health dimensions in infertile women: a systematic review

**DOI:** 10.1186/s40359-023-01285-1

**Published:** 2023-08-31

**Authors:** Fatemeh Yahyavi Koochaksaraei, Masoumeh Simbar, Mehrnoosh khoshnoodifar, Mahbobeh Faramarzi, Malihe Nasiri

**Affiliations:** 1 Student Research Committee, School of Nursing and Midwifery, Shahid Beheshti University of Medical Sciences, Tehran, Iran; 2 Department of Midwifery and Reproductive Health, Midwifery and Reproductive Health Research Center, School of Nursing and Midwifery, Shahid Beheshti University of Medical Sciences, Tehran, Iran; 3 E-Learning Department, Virtual school of Medical Education and management, Shahid Beheshti University of Medical Sciences, Tehran, Iran; 4 Infertility and Reproductive Health Research Center, Health Research Institute, Babol University of Medical Sciences, Babol, Iran; 5 Population and Family Spiritual Health Research Center, Health Research Institute, Babol University of Medical Science, Babol, Iran; 6 Department of Basic Sciences, School of Nursing and Midwifery, Shahid Beheshti University of Medical Sciences, Tehran, Iran

**Keywords:** Infertility, Mental interventions, Mental health, Stress, Anxiety, Depression, Women

## Abstract

**Background:**

Scientific developments have brought hope to infertile couples; however, the results are not always favorable. This makes women suffer psychological pressure. Therefore, previous studies have investigated the effectiveness of many psychological interventions but no research identified the most common psychological interventions. In this regard, the present review aimed to investigate different psychological interventions that promote mental health in infertile to identify the most frequent (common) ones.

**Methods:**

In the present study, the search was carried out using appropriate keywords Infertility, psychological interventions, mental health, stress, anxiety, depression and women in the Google Scholar، Magiran، SID، Pubmed، Scopus، Science Direct، ProQuest، Web of Science databases and One of the leading websites in health- WHO with Persian and English languages and two operators of “AND” and “OR” between 2000 and 2021.

**Results:**

First, 7319 articles were searched, 6948 articles of which were removed due to irrelevant subjects, and 31 articles were removed due to duplication. 340 abstracts were examined and the results of 60 articles were extracted. Two approaches (examining the type and content of intervention) were used to extract findings. The first approach indicated high diversity in psychological interventions, leading to the categorization of the interventions into 4 categories of cognitive behavioral therapy (CBT), mind-body interventions (MBI), stress management skills, and others. CBT and MBI and stress management skills were found as the most frequent promoting interventions for mental health in infertile women. The second approach indicated the differences in protocols (number of sessions and time of interventions).

**Conclusion:**

Despite differences in protocol of interventions under study, the results of all articles revealed the effectiveness of interventions in improving infertile women’s mental health; therefore, it is suggested to apply the most common psychological interventions based on scientific evidence (CBT, MBI, and stress management skills) along with infertility treatment methods. The results will help the specialists, policy-makers, and planners to select and implement the most appropriate psychological interventions for infertile women.

**Supplementary Information:**

The online version contains supplementary material available at 10.1186/s40359-023-01285-1.

## Introduction

According to the World Health Organization (WHO), infertility as one of the world’s health problems has affected millions of people in their reproductive age. About 48 million couples and 186 million individuals suffer from infertility around the world, and infertility-caused disability is the fifth disability in the world [1]. The prevalence of infertility is 15% in the world [1, 2] and it has been reported between 5 and 22% (on average 9.10%) in Iran [3]. In recent years, the prevalence of infertility has been on the rise due to sexually transmitted diseases and environmental pollution [2]. Pregnancy is one of the important goals of developing countries, and infertility is considered a harmful factor for reproductive health, which is associated with many physical and psychological problems [4, 5]. Infertility and treatment for it is a source of mental suffering for infertile women, with direct effects on different dimensions of their mental health (stress, anxiety, and depression) [6, 7]. The results of studies have revealed the reduction of mental health indicators in infertile women so that 44% of infertile women suffer from mental problems and are twice higher at risk of suffering from mental disorders than infertile men [8, 9] and are emotionally more anxious, distressed, and depressed than their husbands [10]. Therefore, it is necessary to identify and use the most common psychological interventions that promote mental health in women with infertility to deal with the psychological consequences of infertility.

The fact that the mental disorders caused by infertility can be prevented and, like other diseases, their implications and chronicity can be mitigated if diagnosed and treated timely can explain the importance of the research [11]. Also, recently, mental health professionals have concluded that psychological interventions are as necessary as medical treatments for infertility. Therefore, it is a need to identify the most common scientific evidence-based psychological interventions to improve mental health and use them along with medical treatments for infertility. In this regard, with the rapid development of assisted reproductive technology (ART), many infertility centers were set up around the world to provide counseling, treatment, and attention to the concerns and emotional needs of infertile patients [12], and many interventional studies have designed and implemented to improve and promote mental health in these patients. However, it should be noted that most interventional studies and even systematic and review studies have only focused on the effects of a single treatment approach (cognitive behavioral, mind-body interventions, educational programs, psychotherapist programs, and counseling) [13–19], and no review or systematic study has investigated a large range of psychological interventions that promote mental health in women with infertility.

Considering the above-mentioned issues, there are various and complex psychological interventions on the issue of infertility; however, no research identified the most common (frequent) psychological interventions in this regard. The present study is an attempt to address this gap for the first time. Regarding the results of previous studies that indicated the importance of performing appropriate psychological interventions along with supporting patients with infertility [20], the present study aimed to systematically review different types of psychological interventions that promote mental health in infertile women to determine the most common psychological interventions used for women with infertility.

## Materials and methods

**Fig. 1:**
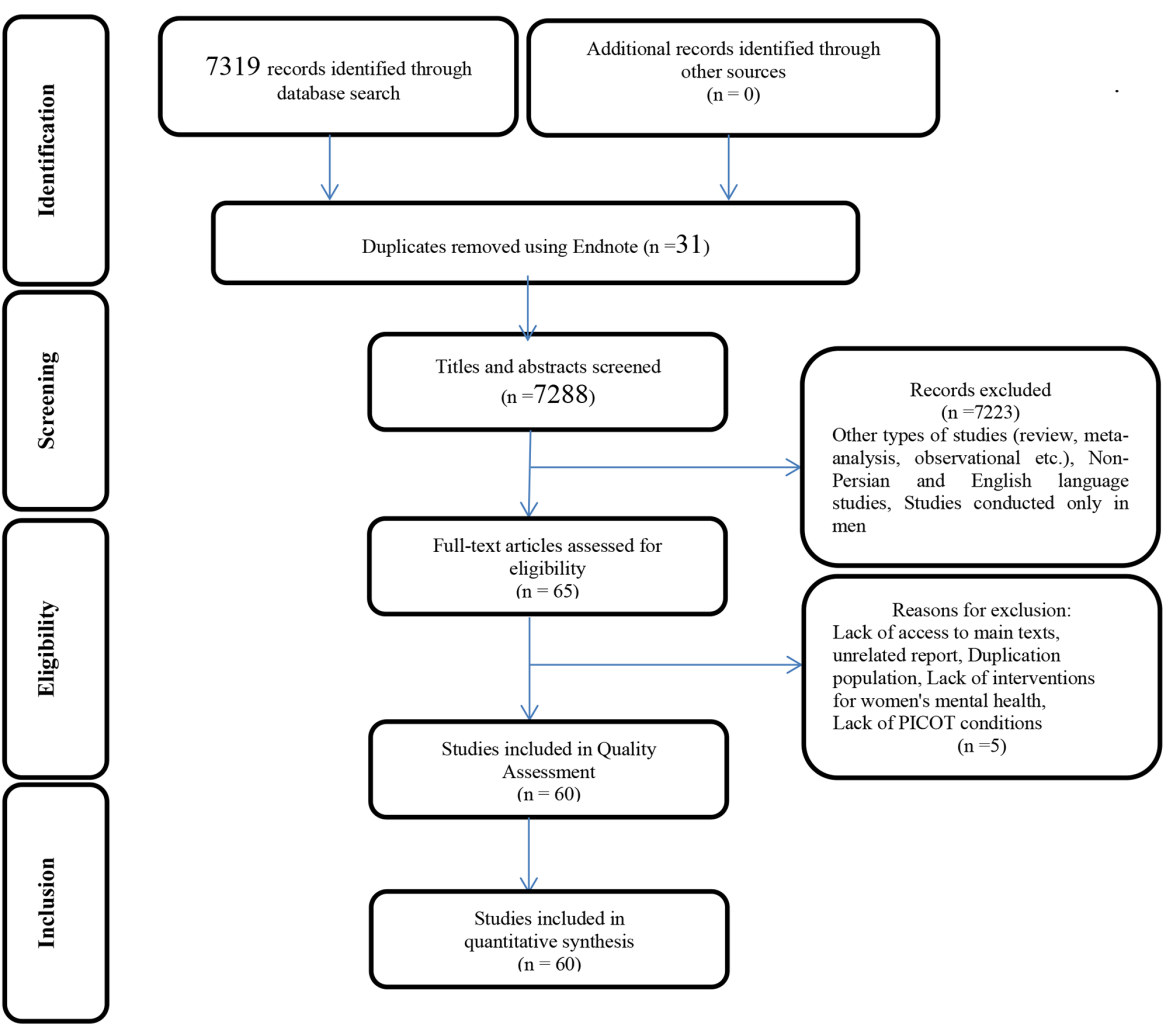
PRISMA flowchart of study entry and exit process

This study was carried out based on the PRISMA (Preferred reporting items for systematic reviews and meta-analyses) systematic review and meta-analysis checklist (Fig. 1). A comprehensive and systematic search was carried out on articles published between 2000 and 2021. The keywords taken from MeSH included infertility, psychological interventions, mental health, stress, anxiety, depression, and women. The databases included Google Scholar, Magira, SID, Pubmed, Scopus, Science Direct, ProQuest, Web of Science, and One of the leading websites in health- WHO. The search was carried out in Persian and English using two operators “AND” and “OR”; for the keywords of the same category, the operator “OR” was used, and “AND” was used to combine the words. Then, considering the following 4 criteria, the articles were selected and the present systematic review was compiled. The criteria included (1) determining the research questions, (2) searching the databases to select articles, (3) selecting articles, and (4) drawing diagrams and Table [21].

To determine the research question, the PICO format was first determined. Then, the research question, i.e. (what are the most frequent (common) psychological interventions to improve mental health in women with infertility?) was typed to be searched.

P (Patient): Infertile women.

I (Intervention): Interventions that promote different dimensions of mental health (stress, anxiety, and depression).

C (comparison): women with healthy fertility.

O (outcomes): mental health.

To search the databases, the researchers used the keywords taken from MeSH, including infertility, psychological interventions, mental health, stress, anxiety, depression, and women to retrieve the related articles for 4 weeks from 30 April to 31 March 2022. Using a manual search, the researchers reviewed the reference list of the retrieved articles to find more articles.

To select the articles, two authors independently evaluated the articles based on the inclusion and exclusion criteria. The inclusion criteria included infertile women, Persian or English articles, articles published in domestic or international journals, articles that examined the effectiveness of psychological interventions on the mental health, anxiety, stress, and depression of infertile women and experimental and quasi-experimental studies. The exclusion criteria were lack of access to the full text of articles, articles that examined other factors such as self-efficacy, psychological well-being, etc. in infertile women, articles with a male target group, review and observational articles, and protocols. Any disagreement in selecting articles was solved by negotiation and the help of a third person in the next step.

The Effective Public Health Practice Project (EPHPP) tool was used to assess the quality of the articles. It is a general instrument to assess different types of interventional studies in six categories: selection bias, study design, confounders, blinding, data collection practices, withdrawals, and dropouts. Once the assessment was fulfilled, each examined practice received a mark ranging between “weak” (1) “moderate” (2), and “strong” (3). The total score was obtained by calculating the average score of each study. The maximum average total score in each study was 3.00. According to the average total score, the quality of the articles was categorized as weak (1.00-1.50), medium (1.51–2.50), or strong (2.51-3.00) [22]. To prevent bias, searching the articles and assessing their quality was performed by two researchers.

To select the articles, two authors independently evaluated the titles, abstracts, and full texts of the articles were first examined. Then, the articles were checked concerning the inclusion and exclusion criteria. The full texts of articles were read and the information (authors’ names, publication year, type of intervention, the content of the intervention, number of sessions, results, main findings, and conclusion) were extracted. Then, the findings were organized into different categories to conduct a review study and answer the research question. The information was shown in 4 tables (interventions related to cognitive-behavioral treatment (Table 1), interventions related to mind-body interventions (Table 2), interventions related to stress management skills (Table 3), and other interventions (Table 4), and the PRISMA flowchart (Fig. 1). The protocol of study was not prepared and the review was not registered.

**Table 1:** General characteristics of studies that were performed by CBT (N = 19)

Authors, year [Reference]	Type of intervention	Intervention protocol	Outcome	Results	Conclusion	Quality assessment EPHPP
Neisi et al., 2012 [23]	Cognitive-behavioral-religious	8 sessions training with homework	Mental health	F = 7.18, Eta = 0.52, P = 0.001	Cognitive-behavioral religious group intervention was effective in increasing mental health (P < 0.001).	Moderate
Gharaei. V et al., 2003 [4]	Cognitive behavioral training	Individual and face-to-face training for 15–20 days	Anxiety	State anxiety Pre, while, post Mean(SD) = 48.6(13.5), 35.6(10.6), 39.8(9.5), pre-while studying p = 0.002, pre-post p = 0.001 Trait anxiety Pre, while, post Mean(SD) = 47.0(10.1), 38.6(8.4), 41.7(7.9), pre-while studying p = 0.000, pre-post p = 0.000, while, post studying p = 0.006	Cognitive behavioral training was effective in reducing anxiety.	Moderate
Oraki et al., 2015 [24]	Cognitive behavioral intervention for anger management	10 sessions of 90 min	Mental health	Pre, post Mean(SD) = 46.84(4.71), 33.53(4.91), F = 29.57, P = 0.001	Training of anger control upon cognitive- behavioral approach caused to increase the mental health in the experimental group.	Moderate
Ashrafian et al., 2019 [25]	Integrative Positive Cognitive Behavioral Therapy	10 sessions of 90 min	Infertility stress and hope	Infertility stress pre, post Mean(SD) = 140.13(2.100), 127.27(4.41), F, p = 77.21, 0.001 Hope pre, post Mean(SD) = 27.47(5.139), 32.20 (5.14), F, p = 77.21, 0.006	Intervention was effective in reducing infertility stress and increasing the hope of infertile women (P < 0.001).	Strong
Kraaij et al., 2015 [26]	cognitive behavioral Self-Help Program (CBS)	4 days a week ,1 h per day for a period of 4 weeks	Depression	Baseline-first posttest F, Eta = 15.53, 0.28, P = 0.00 Baseline-second posttest F, Eta = 9.32, 0.20, P = 0.00	CBS was effective in improving the depression score and its positive effect remained in the follow-up period.	Strong
Manochehri et al.,2005 [27]	Cognitive-emotional-behavioral group counseling	10 sessions of 1.5 h	Mental health	Mental health pre, post, fallow up 1, 2 Mean(SD) = 130.85(59.35), 63.7(28.68), 56.85(17.62), 62.14(23.02), F = 14.43,P = 0.01	The intervention was effective in improving mental health and its effect continued until 3 months after the intervention.	Moderate
Mosalanejad et al., 2012 [18]	Cognitive behavioral therapy	1 h and 30 min weekly session’s group therapy in 15 week	Stress, anxiety, depression, hardiness	Depression pre, post, Mean(SD) = 13.11(4.76), 6.41(3.26), P = 0.001 Anxiety pre, post, Mean(SD) = 11.11(4.45), 7.17(3.84), P = 0.007 stress, pre, post, Mean(SD) = 14.64(4.07), 6.7(4.22), P = 0.001	Significant differences was in level of stress (p = 0.000), anxiety (p = 0.001) and depression (0.007) in treatment group pretest with posttest.	Moderate
Faramarzi et al., 2013 [28]	Cognitive Behavioral Therapy (CBT) and Pharmacotherapy	CBT group (gradual relaxation training, restructuring, and eliminating of negative automatic thoughts and dysfunctional attitudes to infertility for 10 sessions) Antidepressant therapy (20 mg fluoxetine daily for 90 days)	Infertility Stress	CBT Infertility Stress pre, post Mean(SD) = 3.5 (0.62), 2.7 (0.62) p < 0.05 fluoxetine Infertility Stress pre, post Mean(SD) = 3.5 (0.53),0.3.2 ( 4.4) p < 0.05	Both methods significantly reduced the Infertility Stress but CBT was superior to fluoxetine in resolving and reducing of infertility stress.	Strong
Faramarzi et al., 2008 [29]	Psychological intervention, psychotropic medication	CBT groups (10 sessions on relaxation training, restructuring, and eliminating negative automatic thoughts and dysfunctional attitudes to infertility ), antidepressant therapy (20 mg fluoxetine daily for 90 days)	Mental health, Depression	Fluoxetine mental health pre, post, Mean(SD) = 26.7 (11.9), 18.2 (8.8), P = 0.002 CBT pre, post, Mean(SD) = 28.5 (10.3) 13.6 (7.1), P < 0.001 fluoxetine Depression pre, post, Mean(SD)= 23.2 (8.6), 14.3 ( 8.5) ,p < 0.001 CBT Depression pre, post, Mean(SD) = 20( 7.9), 7.7 ( 4.8), p < 0.001	Both methods significantly reduced the mental health and Depression scores, the decrease in the CBT group was significantly greater than the fluoxetine group.	Moderate
Faramarzi et al., 2008 [30]	Cognitive behavioral therapy and fluoxetine	CBT group (gradual relaxation training, restructuring, and eliminating of negative automatic thoughts and dysfunctional attitudes to infertility for 10 sessions), Antidepressant therapy (20 mg fluoxetine daily for 90 days)	Depression, anxiety	Fluoxetine depression pre, post, Mean(SD) = 23.2(8.6), 14.3(8.5), P < 0.001 CBT depression pre, post, Mean(SD) = 20.1(7.9), 7.7(4.8), p < 0.001	Both methods significantly reduced depression and anxiety CBT was superior to fluoxetine in the resolution or reducing of depression and anxiety.	Moderate
Ahmadali Noorbala, 2008 [31]	Psychiatric interventions(cognitive-behavioral therapy, supportive psychotherapy, fluoxetine)	Group 1 (6-month psychological treatment with cognitive-behavioral therapy (CBT), supportive psychotherapy, and 20 to 60 mg per day of fluoxetine	Depression	Depression Pre, post Mean(SD) = 18.7(9.7) to 10.7(5.8), P < 0.001	Depression was significantly lower in group 1 than in group 2 (P < 0.001).	Moderate
Ahmadali Noorbala, 2008 [32]	Pharmacotherapy and psychotherapy	Treated with fluoxetine based on severity of disease, 6–8 session cognitive-behavioral therapy and supportive psychotherapy for six months)	Depression	Depression Pre, post Mean(SD) = 18.7 (9.7), 10.7 (5.8), p < 0.0001	Psychiatric interventions (pharmacotherapy and psychotherapy) was effective in reduction of depression symptoms (p < 0.0001).	Moderate
Heydari. P et al., 2002 [33]	Cognitive behavioral therapy	CBT for 12–13 days	Anxiety	State anxiety Pre, post Mean(SD) = 46.4(9.9), 31.6(8.1), P = 0.0001 Trait anxiety Pre, post Mean(SD) = 47.1(11.1), 40.6(9.9), P = 0.001	Cognitive-behavioral therapy was effective in reducing anxiety.	Moderate
Talaei. A et al., 2014 [34]	Cognitive behavioral group therapy	10 Sessions of 120 min for 2/5 months	Depression	Beck depression Pre, post Mean(SD) = 20(7.37), 14.5(6.54), P < 0.05 Hamilton depression Pre, post Mean(SD) = 21.9(7.23), 16.0(10.83), P < 0.05, F = 35.37	Cognitive behavioral Group therapy was effective in improving depression (p < 0.001).	Moderate
Starabadi et al.,2020 [35]	Cognitive- Behavioral Therapy	10 sessions of 90 min	Infertility Stress and Depression	Infertility Stress Pre, post Mean(SD) = 50.43(16.10), 134.70(14.92), F = 38.22, P = 0.0001 Depression Pre, post Mean(SD) = 29.66(6.12), 21.30(4.56), F = 67.27, P = 0.0001	CBT was effective in decrease of infertility stress and depression.	Strong
Mosalanejad et al., 2012 [36]	E-Cognitive Group Therapy with Emotional Disclosure	Weekly 12-hour meeting for three months), painting sessions (art therapy) and written and verbal emotional disclosure ,individually and in group	Depression, Anxiety, Stress	Depression Pre, post Mean(SD) = 14 (2.38), 8 (2.62), F, P = 000, 0.99 Anxiety Pre, post Mean (SD) = 13.96 (2.59), 8. 06 (2.63), F, P = 2. 9 4, 0.09 Stress Pre, post Mean (SD) = 13.93 (3.15), 8. 84 (2.65), F, P = 10.32, 002	Psychological intervention lowered the level of Depression, Anxiety, Stress; the mean difference between two groups was significant (p = 0.001).	Moderate
van Dongen et al., 2016 [37]	E-Therapy	CBT and usual care and digital psycho-education	Depression, anxiety	Risk difference (95%CI) = 24% (2–46%), P = 0.03	E-Therapy was effective to reduction in the percentage women having clinically relevant symptoms of anxiety, depression in intervention group compared with the control group 3 months after the first ART cycle.	Moderate
Minden B et al., 2010 [38]	Web-based treatment	web-based approach to providing a cognitive behavioral intervention (modules Included cognitive restructuring, relaxation, and behavioral activation to commonly endorsed symptoms and experiences of infertile individuals)	General and infertility-related psychological stress	General stress pre, post Mean(SD) = 5.0(1.3), 5.1(1.1) F(intervention condition and time) = 6.045, p = 0.02	Online cognitive behavioral approach was effective to reduced general stress.	Moderate
Haemmerli et al., 2010 [39]	Internet-based support	8-week (13 sessions) Internet-based cognitive-behavioral treatment	Mental health, pregnancy rate	CES-D pre, post, Mean(SD) = 16.7 (11.7), 11.8 (8.1), D (Between group) = 0.337 State anxiety pre, post, Mean(SD) = 41.4 (11), 36.7 (9.3), D (Between group) = 0.383 Trait anxiety pre, post, Mean(SD) = 41.7 (9.8), 37.8 (9.5), D (Between group) = 0.338 IDS pre, post, Mean(SD) = 25.2 (3.9), 21.6 (5.3), D (Between group) = 0.163	The intervention significantly reduced the depression level of clinically distressed and depressed participants but no effects on pregnancy rate.	Moderate

EPHPPa: Effective Public Health Practice Project, d = Cohen’s d effect size, D: Difference between the Pre- and Post-measures, MD: mean difference, CES-D center for epidemiologic studies depression scale, IDS infertility distress scale

**Table 2:** General characteristics of studies that were performed by MBI (N = 6)

Authors, year [Reference]	Type of intervention	Intervention protocol	Outcome	Results	Conclusion	Quality assessment EPHPP
Bai et al., 2019 [40]	Two guided self-administered interventions (mindfulness, gratitude)	BMG (a weekly session of 1 h for 4 weeks and at least 20 min of daily practice at home), GJG (a weekly session of 1 h for 4 weeks and the three gratitude journals exercise daily at home), CG (routine care)	Depression, anxiety, sleep quality, infertility-related stress, mindfulness and gratitude	Depression mean difference (95% CI) = − 1.69(− 3.01 to − 0.37), *d* = 0.44 Sleep quality mean difference (95% CI)= -1.24, [− 1.95, − 0.39], *d* = 0.43	The brief mindfulness intervention was effective in reducing depression and improving sleep quality in BMG (p = < 0.001).	Moderate
Clifton et al., 2020 [16]	Internet-based mind/body intervention	10 sessions of 1 h equivalent to 10 face-to-face sessions	Distress (anxiety and depression)	BDI B, d= -7.98, -0.86, p = 0.01 BAI B, d= -4.86, -0.67, p = 0.00 PSS B, d= -4.15, -0.61, p = 0.08 FPI Total B, d= -6.14, -0.12, p = 0.60	In the intervention group, anxiety (P = 0.003), depression (P = 0.007), perceived stress (P = 0.041), fertility-social (P = 0.018), fertility-sexual (P = 0.006) decreased significantly.	Strong
Galhardo et al., 2013 [41]	Mindfulness-Based Program	10 weekly sessions, group format, 2 h (men attend three Sessions)	Depression, state anxiety, entrapment, defeat, internal and external shame, experiential avoidance, mindfulness, self-compassion, and infertility self-efficacy	Depressive Pre, post Mean(SD) = 11.02(7.05), 6.18(4.05), t = 5.46, p = 0.001 External shame Pre, post Mean(SD) = 8.73 (8.72), 5.85 (6.60), t = 3.62, p = 0.001 Internal shame Pre, post Mean(SD) = 6.78 (6.35), 4.13 (4.86), t = 3.20, p = 0.002 Entrapment Pre, post Mean(SD) = 55.40 (17.84), 48.33 (14.91), t = 3.95, p < 0.001 Defeat Pre, post Mean(SD) = 19.05 (10.67), 14.49 (8.24), t = 3.37, p = 0.001 Self-efficacy Pre, post Mean(SD) = 81.87 (24.45), 98.87 (19.58), t = 6.00, p < 0.001	MBPI was effective in decrease of depressive symptoms, internal and external shame, entrapment, and defeat and increase in mindfulness skills and self-efficacy to deal with infertility.	Moderate
Chan, C.H et al., 2006 [42]	Eastern Body-Mind-Spirit (EBMS) group intervention	4 Sessions of 3 h, weekly, group counseling	Anxiety	State anxiety score T1, T2, T3 = 47.12, 43.53, 42.69, F _T1, T2, T3_ = 4.663, P < 0.01	In the intervention group, the Eastern body-mind-spirit group intervention was effective in reducing anxiety (P < 0.01).	Moderate
Kalhori et al., 2020 [17]	Mindfulness-Based Group Counseling	Eight 90-minute sessions (two each week)	Depression	Depression pre, post Mean(SD)= 20.77 ( 6.35), 10.82 (7.16), P < 0.001	Mindfulness-based group counseling was effective to reduce depressive symptoms.	Strong
Psaros et al., 2014 [43]	Mind–body group treatment	10-week group program (The groups met once weekly for 2 h with one weekend 4-h sessions)	Psychological variables(Depression, MOS, PSS, LOT-R) salivary cortisol levels	Depression pre, post Mean(SD) = 17.9 (1.2), 10.1 (1.3), t = 4.85, p < 0.001 PSS pre, post Mean(SD) = 8.5(0.4), 6.1 (0.5), t = 4.79, P = 0.001 LOT-R pre, post Mean(SD) = 10.7 (0.9), 9.9 (0.9), P = 0.660 MOS social support pre, post Mean(SD) = 68.9 (2.8), 80.4 (3.0), t = 4.98, p = 0.001	MBI was effect to decrease of depression and perceived stress and increased perceived social support but there were no significant changes in cortisol levels.	Strong

EPHPP^a^: Effective Public Health Practice Project, BDI = Beck’s Depression Inventory-II; BAI = Beck’s Anxiety Inventory; PSS = Perceived Stress Scale; FPI = Fertility Problem Inventory; d = Cohen’s d effect size, D: Difference between the Pre- and Post-measures, MD: mean difference, MOS Social Support Survey (emotional information support subscale), Life Orientation Test-Revised (LOT-R), Perceived Stress Scale (PSS-4), brief mindfulness group (BMG), gratitude journal group (GJG), control group (CG).

**Table 3:** General characteristics of studies that were performed by SMS (N = 5)

Authors, year [Reference]	Type of intervention	Intervention protocol	Outcome	Results	Conclusion	Quality assessment EPHPP
Hashemi. F et al., 2013 [44]	Stress management skills	Group training during 10 sessions of 2 h per week	Mental health	F = 74.63, Eta = 0.780, P = 0.000	The mental health of the experimental group increased in the post-test phase (P < 0.05).	Moderate
Koumparou et al., 2021 [45]	Stress management	8 weekly Stress management sessions	Depression, Anxiety, Stress, Perceived Stress, Fertility Problem Inventory	Perceived stressT1,T2 Mean(SD) = 26.5 (8.1), 18.6 (7.1), p < 0.001 Depression T1,T2 Mean(SD) = 6.3 (6.33), 1.76 (2.97), p < 0.001 Anxiety T1,T2 Mean(SD) = 4.66 (5.43), 1.7 (3.17), p < 0.001 Stress T1,T2 Mean(SD) = 7.85 (5.98), 3.38 (4.29), p < 0.001 Global stress T1,T2 Mean(SD) = 148 (38.1), 131.9 (29.6), p < 0.001	Total stress in the intervention group declined significantly (p < 0.001) in respect to all the parameters of the PSS-14, DASS-21 and FPI scales.	Strong
Hamid N, 2011 [46]	Stress Management	Intervention group (12 sessions of 2 h) control group (did not receive intervention)	Depression, Anxiety and Fertilization	Depression pre, post, fallow up Mean(SD) = 60.21(8.41), 26.18(5.91), 26.09(5.61), F = 9.16, P = 0.001 Anxiety pre, post, fallow up Mean(SD) = 20.32(4.83), 14.17(2.12), 13.92(2.17), F = 8.56, P = 0.001	Stress management training was effective in reducing and anxiety in the post-test phase and 12-month follow-up.	Strong
Heredia et al., 2019 [47]	Psychological intervention focused on stress management	A 90-minute session includes psychoeducation, relaxation and coping skills	State anxiety, emotional imbalance, perceived quality of life	State anxiety D, F = − 3.90, 9.69, P = 0.005 Emotional imbalance D, F = − 17.50, 4.90, P = 0.037 QoL Total D, F = 10.00, 4.27, P = 0.049	Psychological intervention focused on stress management was effective in reducing anxiety and emotional imbalance and improving perceived quality of life.	Moderate
Akiko MORI, 2009 [48]	Supporting stress management	Experimental group (asked to continue stress management homework for 3 months)	Risk ratio of depression and anxiety, health status	Anxiety risk ratio(95% CI) = 1.05( 0.54–2.04) Anxiety baseline, At 1, 2, 3 month Mean(SD) = 5.5(3.19), 5.5 (3.88), 5.2 ( 3.55), 4.5( 3.90), p = 0/021 Depression risk ratio(95% CI) = 1.17 ( 0.55–2.47) Depression baseline, At 1, 2, 3 month Mean(SD) = 4.2 ( 2.64), 10.2 ( 6.59), 4.6 (3.27), 4.4 ( 3.46), p = 0.018 Role functioning physical F (time × program)= 10.52, *P* = 0.002 physical component summary F (time ×program) = 12.68, P = 0.001	Support did not prevent the incidence of depression and anxiety but Positive effects were observed on role functioning physical and physical component summary.	Moderate

EPHPP^a^: Effective Public Health Practice Project, d = Cohen’s d effect size, D: Difference between the Pre- and Post-measures, MD: mean difference, quality of life (QoL).

**Table 4:** General characteristics of studies that were performed by other interventions (N = 30)

Authors, year [Reference]	Type of intervention	Intervention protocol	Outcome	Results	Conclusion	Quality assessment EPHPP
Gojani et al., 2017 [49]	Problemsolving skill training (PSS)	3 sessions of 40 to 45 min	Mental health and success of treatment	State Anxiety mean changes = − 6.41(12.11), *t* = 0.308, *P* = 0.004 Trait anxiety mean changes = 5.26(8.72), *Z* = 3.06, *P* = 0.002 Depression mean changes = 5.55(6.15), *t* = 5.266, *P* < 0.001	PSS was effective in reducing anxiety and depression but success of treatment was not effective (P = 0.230).	Strong
Bahrami Kerchi et al., 2020 [50]	Psychological Empowerment Package and Dialectical Behavior Therapy	Psychological empowerment package (11 sessions of 90 min) and dialectical behavior therapy (8 sessions of 90 min)	Depression	F = 198.23, Eta = 0.82, P < 0.001	Empowerment package was effective in reducing depression (P < 0.001).	Moderate
Nekavand M et al., 2015 [51]	Relaxation	3 sessions of 1 h of group intervention	Infertility anxiety	F = 3.33, Eta = 0.033, P = 0.001	Relaxation was effective in reducing anxiety in experimental group (P < 0.05).	Moderate
Khalatbari et al., 2018 [52]	Integrated model of emotional focused approach and Gottman model	10 sessions of 120 min group intervention	Fear of Intimacy and anxiety	Fear of Intimacy F, Effect size = 27.09, 0.60, P = 0.001 Anxiety F, Effect size = 82.36, 0.60, P = 0.001	Both methods were effective in reducing fear of intimacy and anxiety (P < 0.001).	Moderate
Hosseini, M et al., 2017 [53]	Holistic-oriented psychological	11 sessions of two-hour (10 sessions for infertile women and 1 session for husbands)	Depression Anxiety Stress	Depression F, Eta = 30.33, 0.551, p = 0.000 Anxiety F, Eta = 36.59, 0.602, p = 0.0001 Stress F, Eta = 38.10, 0.604, p = 0.0001	Holistic-oriented psychological was effective in reducing anxiety, depression and stress (p < 0.0001).	Moderate
Hosseini, M, 2018 [54]	Holistic-oriented psychological	11 sessions of 120 min	Psychological health and fertility rate	Depression F, Eta = 42.19, 0.63, p < 0.001 Anxiety F, Eta = 40.47, 0.602, p < 0.0001 Stress F, Eta = 34.58, 0.58, p < 0.0001	Holistic-oriented psychological was effective in reducing anxiety, depression and stress in the post-test phase (p < 0.001).	Strong
Rahimi, R et al., 2015 [55]	Psychodrama	10 sessions of 2 h (one session per week)	Depression	F = 42.83, Eta = 0.611, P = 0.001	Psychodrama was effective in reducing depression (p < 0.001).	Moderate
Gojani et al2017, [56]	Positive reappraisal and problem-solving skills training	Positive reappraisal n (2 sessions) and problem solving skills (3 sessions) and control (routine care)	Anxiety	Positive reappraisal F = 10.75, p = 0.001, problem-solving skills F = 4.58, p = 0.018 control group F = 4.49, p = 0.015	Anxiety decreased in both training groups and increased in the control group.	Strong
Farnia et al., 2019 [57]	Nursing versus peer-based education methods	Nurse and peer-educated groups(30–60 min group education) Control (Only pre-discharge education)	Anxiety	Nurse-educated anxiety pre, post Mean (SD) = 44.47 ( 11.12), 39.38 ( 11.08), p < 0.001 Peer-educated pre, post Mean (SD) = 46.92 (9.87), 41.06 ( 9.27), p < 0.001	Both nurse and peer education programs were effective in reducing preoperative anxiety.	Moderate
Matthiesen et al., 2012 [58]	Expressive writing intervention (EWI)	Intervention group (three EWI writing tasks of 20-minute duration conducted over a 3-day period), control group (write in an emotionally neutral manner about their daily activities)	Infertility-related stress	Infertility stress pre, post, Follow-up Mean(SD) = 14.73 (8.7), 10.8 (8.23), 14.6, (8.63) Effect time F, Eta = 2.28,0.32, P = 0.005 Interaction (group × time) F, ETA = 2.28 ,0.17, *p* = 0.07	Infertility-related stress was decreases after the intervention in the EWI group compared to controls.	Moderate
Zaidouni et al., 2019 [59]	Nursing Consultation Based on Orem’s Theory of Self‑care and Bandura’s Concept	3 sessions of 1 h each, 1 week apart	Infertility Stress	Perceived stress Pre, post Mean(SD) = 32.96 (1.819), 25.07 (6.19), *t* = 9.426, *P* < 0.001 General self‑efficacy stress Pre, post Mean(SD) = 22.59 (5.396), 29.23 (5.743), *t*= ‑6.888, *P* < 0.001	Nursing consultation was effective to decreasing perceived stress and increased self‑efficacy.	Moderate
Rabeipour et al., 2016 [60]	Group counseling by collaborative approaches	10 sessions of group counseling, including infertility treatment strategy, stress management, problem solving	Stress	Stress pre, post Mean(SD) = 170.52(30.71), 137.86(34.76), P = 0.002	Group counseling with collaborative approach was effective in reducing the stress of infertility.	Strong
Latifnejad Rodsarei et al., 2011 [61]	Infertility collaborative counseling	Five individual sessions, counseling with the participation of a midwife, a gynecologist and a clinical psychologist	Perceived stress	Perceived stress pre, post Mean(SD) = 26.8(153.6), 25.7(144.7), df = 28, P = 0.004	Collaborative counseling was effective in reducing the Perceived stress.	Moderate
Hamzehgardeshi et al., 2019 [14]	Group counseling	6 sessions of 2 h for 6 weeks	Perceived stress	Perceived stress pre, post T1, 2, 3 Mean(SD) = 166.75 ( 13.27), 115.75 ( 13.88), 118.08 (15.37), 120.50 ( 16.24), p < 0.001	Group counseling was effective in reducing the Perceived stress.	Moderate
Rahimi et al.,2021 [62]	Hope-oriented group counseling	Six 45–60 min sessions (once a week)	Mental health, quality of life (QoL)	Stress adjusted mean difference (95% CI) = − 1.7(− 3.2 to − 0.3), P = 0.018 Depression adjusted mean difference (95% CI) = − 1.3( 4.7 to − 1.5), P < 0.001 QoL adjusted mean difference (95% CI) = 6.9 (5.1 to 8.8), P < 0.001	Hope-oriented group counseling was effective in reducing stress and depression and improving (QoL).	Moderate
Mokhtari Sorkhani et al., 2021 [63]	Counseling for Infertile Couples	6 sessions of 45 min twice a week	Women’s Emotional Disturbance	Depression Mean(SD) = 1.55(1.92), P < 0.0001 Social support Mean(SD) = 15.73(3.41), P < 0.0001 Cognitions regarding fertility difficulties Mean(SD) = 26.48(3.05), P = 0.001 Anxiety Mean(SD) = 25.03(3.09), p = 0.35	Infertility counseling improved the domains of infertile women’ emotional status except, anxiety.	Moderate
Jamshidian-Qalehshahi et al., 2017 [64]	Iranian–Islamic Positive Therapy	11 sessions of 90 min	Depression, Anxiety, Stress	Depression Pre, post, fallow up Mean(SD) = 23.08(3.27), 16.69(4.19), 18.08(5.39), Post, fallow up F = 0.39, 0.26, Post, fallow up P = 0.0001, 0.010 Anxiety Pre, post, fallow up Mean(SD) = 16.07(2.07), 11.23(2.68), 12.07(3.06), Post, fallow up F = 0.52, 0.37, Post, fallow up P = 0.0001, 0.001 Stress Pre, post, fallow up Mean(SD) = 24.92(4.54), 18.38(2.99), 19.07(4.27), Post, fallow up F = 0.54, 0.40, Post, fallow up P = 0.0001, 0.001	Iranian–Islamic Positive Therapy is effective in reducing depression, anxiety, and stress in posttest, short- and long-term follow up.	Moderate
Zarif Golbar Yazdi. H et al., 2012 [65]	Wellbeing therapy	8 sessions weekly	Stress and psychological well-being	Stress Pre, post Mean(SD) = 25.81 (3.15), 14.54 (4.48), p < 0.026 Wellbeing Pre, post Mean(SD) = 297.72 (32.64), 383.45 (35.02), p < 0.000	Wellbeing therapy was effective in reducing stress and increasing psychological well-being (p < 0.05).	Moderate
Shahi-Senobari S et al., 2021 [66]	Positive psychotherapy based on belief to good	8 group sessions of 90 min, weekly	Mental health and marital quality	Mental health Pre, post, fallow up Mean(SD) = 30.73(3.75), 22.4(4.61), 22.4(3.99), F (Time× Group) = 30.1, P = 0.00 Marital quality Pre, post, fallow up Mean(SD) = 40.46(5.99), 48.73(6.43), 47.06(5.43), F (Time× Group) = 39, P = 0.000	Intervention was effect on mental health (P > 0.01), marital quality (P > 0.01and mental health outcomes were stable up to one-month follow-up (P > 0.01).	Moderate
Bahrami Karchi. A et al., 2020 [50]	Psychological empowerment therapy and dialectical behavior therapy	Psychological empowerment (11 sessions of 90 min), Dialectic behavior therapy (8 sessions of 90 min)	Infertility stress	Mean difference(MD)= -41.62 F = 554.74, Eta = 0.864, P = 0.001	Psychological empowerment therapy was more effective in reducing infertility stress than Dialectical Behavior Therapy.	Strong
Jamshidian QalehShahi et al.,2017 [67]	Iranian Positive Therapy(IPT) and Acceptance -Commitment Therapy(ACT)	11 sessions of 90 min, weekly	Depression, Anxiety, Stress	IPT Depression Pre, post, fallow up Mean(SD) = 23.08(3.27), 16.69(4.19), 18.08(5.39) ACT Depression Pre, post, fallow up Mean(SD) = 22.85(3.07), 16.31(5.21), 18.15(5.11), Group F, Eta, p = 7.36, 0.285, 0.002 IPT Anxiety Pre, post, fallow up Mean(SD) = 16.38(2.21), 11.15(2.68), 12.07(3.06) ACT Anxiety Pre, post, fallow up Mean(SD) = 16.07(2.07), 11.23(2.37), 11.46(4.43), Group F, Eta, p = 12.94, 0.412, 0.001 IPT Stress Pre, post, fallow up Mean(SD) = 24.92(4.54), 18.38(2.99), 19.07(4.27) ACT Stress Pre, post, fallow up Mean(SD) = 25.23(4.19), 18.77(3.9), 18.92(4.31), Group F, Eta, p = 8.86, 0.32, 0.001	Both of the therapeutic methods were equally effective in reducing anxiety, stress and depression.	Moderate
Pasha et al., 2018 [68]	Antidepressant medication and psychological intervention	Psychosexual therapy(PST): weekly 2-hour session for 8 times, bupropion extended-release (BUP ER) at a dose of 150 mg/d for 8 week	Depression	PST Pre, post Mean(SD) = 24.59 (7.76), 10.42 (9.01), BUP ER Pre, post Mean(SD) = 22.42 (10.70),16.09 ± 11.81, Group F, P = 8.153, 0.001	PST can greater improvement in depression levels in comparison with the two other groups (P < 0.001).	Moderate
Soltani et al., 2014 [69]	Emotionally Focused Therapy (EFT)	10 sessions emotionally focused couple therapy program	Depression, Anxiety, Stress	Depression Mean differences (SD) = 3.83(2.03), Z= -3.58, p < 0.0001 Anxiety Mean differences (SD) = 6.0(3.01), Z= -3.49, p < 0.0001 Stress Mean differences (SD) = 6.91(3.7), Z= -4.18, p < 0.0001	EFT decreased the rate of depression, anxiety in experimental group in comparison with the control group (p < 0.0001).	Moderate
de Barros Fleury et al., 2021 [70]	Music Therapy	50-minute individual sessions were applied before baseline ultrasound scan, oocyte pick-up, and embryo transfer.	Stress	Stress Pre, post, Mean(SD) = 20.4(9.91), 16.12(7.87), p = 0.004	Music therapy was effective for stress reduction.	Strong
Aba et al., 2017 [71]	Music therapy	Twenty-eight minutes of music therapy was applied to the music therapy group 1 h before and after the embryo transfer	Anxiety, pregnancy rate	State anxiety Pre, post, Mean(SD) = 38.16 (9.77), 33.39 (7.56), Trait anxiety Pre, post, Mean(SD) = 40.24 (7.29), 38.19 (7.36), p > 0.05	After the two sessions of music therapy, state and trait anxiety levels decreased and pregnancy rates increased (p > 0.05).	Moderate
Mosalanejad, et al., 2013 [72]	Logotherapy	Spiritual group psychotherapy counseling for 12 sessions, 2 h per week for a 3 months	Perceived Stress, worry	Perceived stress Pre, post, Mean(SD) = 29.25(4.75), 28.18 (4.94), p = 0.27 Worry Pre, post Mean(SD) = 33.25(12.24), 27.31(13.50) p = 0.004	Logotherapy was effective to decrease psychiatric symptoms of worry and perceived stress. (p = 0.01)	Moderate
Hughes et al., 2011 [73]	Art therapy	Weekly 2-h art therapy group	Hopelessness, Depression, Anxiety	Hopelessness pre, post Mean(SD) = 6.1 (3.8), 3.5 (3.1), P = 0.01 Depression pre, post Mean(SD) = 19.8 (11.0), 12.5 (10.2), P = 0.01 Anxiety pre, post Mean(SD) = 12.4 (8.4), 8.4 (5.2), P = 0.3	Art therapy was effect on decrease levels of hopelessness and depressed mood.	Moderate
Cousineau et al., 2007 [19]	Online psychoeducational support	View the program for two 45-min sessions or over several sittings over a four-week period with content up to 90 min	Infertility distress, infertility self-efficacy (ISE), decisional conflict, marital cohesion, coping style	Infertility-related stress (Group 1) pre, post, Mean(SD) = 162.73 (38.04), 153.74 (38.48) Infertility-related stress (Group 2) pre, post, Mean(SD) = 160.31 (34.03), 158.61 (36.07), Z = 1.63, P = 0.10, d = 0.19 ISE(Group 1) pre, post, Mean(SD) = 51.41 (16.92), 56.06 (17.80), ISE(Group 2) pre, post, Mean(SD) = 50.10 (17.46), 52.14 (20.32), Z = 1.68, P = 0.093, d = 0.25	Web-based patient education intervention was effective to decreased global stress and increased infertility self-efficacy.	Strong
Jasani et al., 2016 [74]	Structured Yoga Program	Six-week yoga intervention (Each week included 30 min of discussion, 45 min of gentle Vinyasa-style yoga and 10–15 min of relaxation (savasana)).	Anxiety	State anxiety baseline, follow up Mean = 48.2, 38.4, p < 0.001 Trait anxiety baseline, follow up Mean = 44.6, 39.1, p < 0.014	Anxiety level was significantly lower after a structured six week yoga intervention (p < 0.05).	Moderate
Smith et al., 2011 [75]	Acupuncture	Six sessions of acupuncture over 8 weeks	Infertility self-efficacy, anxiety, infertility-related stress	Social concern MD (95%CI) = − 3.75(− 7.58 to 0.84), p = 0.05 Relationship concern MD(95% CI ) = − 3.66(− 6.80 to 0.052), p = 0.02 Self-efficacy MD (95% CI) = 11.09(− 2.20 to 26.0), p = 0.09 Anxiety MD(95%CI) = − 2.54(− 5.95 to 0.86), p = 0.08	Acupuncture was effective in reducing domains on the infertility stress, anxiety and improving self-efficacy.	Strong

EPHPP^a^: Effective Public Health Practice Project, d = Cohen’s d effect size, D: Difference between the Pre- and Post-measures, MD: mean difference, Perceived Stress Scale (PSS-4).

### Ethical considerations

#### Ethics approval

is not required as the systematic review does not involve the participation of human subjects; rather it involves reviewing and collecting data from publicly available sources. However, the present research was extracted from the doctoral dissertation on reproductive health with code of ethics (IR.SBMU.PHARMACY.REC.1400.011) from Shahid Beheshti University of Medical Sciences.

## Results

First, according to the research question, 7319 articles were found. Then, 6979 articles were removed (6948 articles due to irrelevant titles and 31 articles due to duplicate data). The abstracts of the remaining 340 articles were examined. After reviewing the abstracts, 275 articles were removed and 65 articles remained. Then, the full texts of 65 articles were examined and 5 other articles were removed, and finally, the results of the remaining 60 articles published between 2002 and 2021 were used to write the present review (Fig. 1). To extract the findings from 60 articles, the researchers used two approaches (investigating types and contents of interventions). The results of the first approach indicated high diversity of psychological interventions on women with infertility so that among the articles under study, 19 studies investigated cognitive-behavioral treatment (CBT), 6 articles investigated mind-body interventions (MBI), 5 articles focused on stress management skills (SMS), 3 articles studied collaborative counseling, 3 articles investigated positive psychology (PP), 2 articles examined problem-solving skills, 2 articles investigated music therapy, 2 articles studied holistic psychological interventions (HPI), and 18 articles focused on other interventions (like yoga, acupuncture, relaxation, etc.). On the other hand, this approach led to the organization of psychological interventions into 4 categories of cognitive behavioral therapy, mind-body interventions, stress management skills, and other interventions. Thus, three more common interventions that promote mental health in women with infertility (anxiety, stress, and depression), i.e., cognitive behavioral therapy, mind-body interventions, and stress management skills, were identified. The results of the second approach (investigating the contents of the intervention) indicated the differences in implementation protocols (number of sessions and time), which are discussed in the following.

### Cognitive behavioral therapy (CBT)

19 articles used in this study focused on this technique. The interventions were often in the form of individual or group training sessions; however, they varied in terms of the number of sessions and duration of intervention; 3 studies conducted cognitive behavioral interventions in ten 90-minute sessions, 1 study in 8 sessions, 1 study in 6–8 sessions, 1 study in fifteen 90- minute sessions (15 weeks), 1 study in 12 h per week for 3 months, 1 study in 15–20 days, 1 study in 12–13 days, 1 study conducted the internet-based cognitive behavioral therapy for 8 weeks (13 sessions), 1 study conducted 1-hour sessions 4 days a week for 4 weeks, 1 study conducted ten 120-minute sessions for 2/5 months ,2 study conducted psychotherapy with CBT along with supportive psychotherapy and medication therapy for 6 months, 2 studies conducted an electronic and web-based intervention during the ART cycle and the waiting period after embryo transfer and 3 studies conducted CBT and Pharmacotherapy (20 mg fluoxetine daily for 90 days). The results of all studies indicated that CBT is an effective intervention in changing attitudes and beliefs and can help improve mental health and prevent and reduce anxiety, stress, and depression in women with infertility (Table 1).

The results of studies by Oraki et al. [24], Faramarzi et al. [29], and Manochehri et al. [27] indicated the effectiveness of CBT in improving mental health in infertile women [24, 27, 29]. Starabadi et al. [35], Afshariyan et al. [25], Faramarzi et al. [28], and Minden et al. [38] revealed the effectiveness of CBT in reducing stress caused by infertility. Starabadi et al. found that cognitive-behavioral techniques caused adaptive thoughts in individuals, and training in behavioral methods was an effective treatment to reduce stress and depression caused by infertility [35]. Afshariyan et al. showed that the integrated approach of positive cognitive behavioral therapy was effective in reducing stress caused by infertility and increasing hope, and suggested its application [25]. Faramarzi et al. also confirmed the effectiveness of CBT in reducing infertility compared to fluoxetine [28].

The results of studies by Kraaij et al. [26], Talaei et al. [34], Nisi et al. [23], Mosalanejad et al. [18], Noorbala et al. [31], and Faramarzi et al. [30] indicated the effectiveness of CBT on reducing infertility-caused depression. In studies by Talaei et al. [34], Mosalanejad et al. [36], and Noorbala et al. [32], a significant difference was found between the intervention and control groups in reduced scores of depression in women with infertility after CBT [26, 31, 32, 34, 36]. In the study of Nisi et al., the cognitive behavioral group training reduced depression, which led to a reduction in negative attitudes, and an increase in positive beliefs [23]. Faramarzi et al. also confirmed the effectiveness of CBT in reducing depression and anxiety compared to fluoxetine [30]. The studies of Dongen et al. [37], Qaraei et al. [4], Mosalanejad et al. [18], and Heidari et al. [33] indicated the effectiveness of CBT in reducing infertility-caused anxiety. In the study of Dongen et al., electric therapy programs (online psychological training and CBT) led to a reduced number of women with clinical symptoms of anxiety and depression over 3 months after the first ART cycle compared to the control group [37]. In the study of Qaraei et al., Mosalanejad et al. and Heidari et al., there was a significant difference between the intervention and control groups in the scores of anxiety before and after the study [4, 18, 33].

### Mind body intervention (MBI)

In the present study, 6 articles used this technique. In all of these articles, the interventions were in the form of training sessions; however, there were differences in the number of sessions and duration of interventions; 2 studies investigated mind-body intervention in ten 120-minute sessions, 1 study investigated internet-based intervention in ten 60-minute sessions, 1 study in eight 90-minute sessions, 1 study in four 60-minute sessions along with 20 min of daily practices at home, and 1 study conducted the intervention in four 3-hour sessions. Also, the results indicated the positive effect of MBI in improving mental health and mitigating the symptoms of anxiety, stress, and depression in women with infertility (Table 2). In the study of Clifton et al. [16], the internet-based mind-body intervention reduced distress (anxiety, depression, stress) in women with infertility, and the chance of fertility in the intervention group was 4.47 times more than that in the control group [16]. The results of studies by Kalhori et al. [17], Bai et al. [40], Psaros et al. [43], and Galhardo et al. [41] showed the effectiveness of MBI in reducing infertility-caused depression. In the study of Kalhori et al. group counseling based on mindfulness was effective in reducing depression symptoms in women undergoing IVF [17]. Bai et al. showed that mindfulness not only reduced depression but also improved sleep quality [40]. The results of a study by Psaros et al. indicated the effect of MBI on reducing perceived depression and stress and increasing perceived social support [43]. Galhardo et al. found a significant difference in the score of depression between the intervention and control groups after the intervention so that an increase in mindfulness skills, acceptance, and decentralization led to reduced mental distress in women with infertility [41]. The study by Chan et al. also indicated the effectiveness of the mind-body-spirit intervention in reducing anxiety in women undergoing IVF (In vitro fertilization) compared to women in the control group, and the fertility rate was higher in the intervention group than in the control group [42].

### Stress management skill (SMS)

In the present study, 5 articles investigated the use of this technique in the form of training sessions. However, they varied in terms of the number and duration of the sessions; 1 study investigated the stress management intervention in twelve 120-minute sessions, 1 study in ten 120-minute sessions, 1 study in 8 sessions of a maximum of 60 min, 1 study in one 90-minute session, and 1 study sent the interventions monthly to email addresses of women with infertility for 3 months. The results of the studies indicated the effectiveness of SMS in improving mental health and reducing the symptoms of anxiety, stress, and depression in women with infertility (Table 3).

In the study of Hashemi et al. [44], training stress management skills were found to be effective in increasing mental health in women with infertility [44]. The findings of a study by Koumparou et al. [45] indicated the effectiveness of stress management sessions in reducing the stress of all women undergoing IVF [45]. Akiko et al. [48], Hamid [46]. and Heredia et al. [47] revealed the effectiveness of stress management skills in reducing anxiety and depression. Akiko et al. performed a supportive stress management program on women undergoing infertility treatment and succeeded to reduce anxiety and depression in patients [48]. Hamid found significant differences in the scores of depression and anxiety between the intervention and control groups after the intervention and during a 12-month follow-up process [46]. Heredia et al. found that short-term interventions focusing on stress management can contribute to psychological adjustment in women undergoing IVF, reduce their anxiety levels and improve their quality of life [47].

### Other interventions

In the 4th category, other psychological interventions that promote mental health in women with infertility were examined. The number of retrieved articles related to other interventions was less than that of CBT, MBI, and SMS. 3 articles studied collaborative counseling, 3 articles investigated positive psychology (PP), 2 articles examined problem-solving skills, 2 articles investigated music therapy, 2 articles studied holistic psychological interventions (HPI), and other interventions (yoga, acupuncture, relaxation, etc.) were examined each in one separate study. Table 4 presents the following interventions: Problem-solving skill training (PSS), Nursing versus peer-based education methods, Expressive writing intervention (EWI), Nursing consultation based on Orem’s theory of self‑care and bandura’s concept, Group counseling by collaborative approaches, Infertility collaborative counseling, Group counseling, Hope-oriented group counseling, Counseling for infertile couples, Iranian–Islamic positive therapy, Positive psychotherapy based on belief to good, Psychological empowerment therapy and dialectical behavior therapy, Iranian positive therapy (IPT) and Acceptance-Commitment Therapy (ACT), Antidepressant medication and psychological intervention, Emotionally Focused Therapy (EFT), Music therapy, Logotherapy, Art therapy, Online psychoeducational support, Structured yoga program, Acupuncture, Wellbeing therapy, Positive reappraisal and problem-solving skills training, Psychodrama, Holistic-oriented psychological, Integrated model of emotional focused approach and Gottman model, Relaxation, Psychological empowerment package and dialectical behavior therapy.

## Discussion

The results of this systematic review study indicated a high diversity in the types and protocols of psychological interventions in infertile women. Moreover, the examinations not only led to the categorization of interventions into four categories of cognitive behavioral therapy (CBT), mind-body interventions (MBI), stress management skills (SMS), and other interventions, but also contributed to identifying 3 most common interventions promoting mental health in women with infertility, including CBT (with 19 articles), MBI (6 articles), and SMS (5 articles). It should be noted that despite using different implementation protocols for interventions, the results of all articles indicated the effectiveness of interventions in improving mental health. According to findings, CBT was one of the most frequent interventions (with 19 relevant articles) used for women with infertility. The application of CBT, including various methods of relaxation (muscular and respiratory), cognitive reconstruction, desensitization, behavioral training, thought-stopping, and courage training, has been suggested by all researchers as one of the methods to deal with psychological problems during infertility treatment.

In explaining the findings, it can be said that the events and accidents do not upset individuals but their thinking way that is the result of their attitudes and beliefs and influences the information process and causes emotional reactions in individuals; therefore, one’s thoughts and beliefs about infertility affect the person’s type and level of reaction. Thus, since CBT is a short-term skill-focused psychological approach and aims at changing maladaptive emotional responses through changing thoughts and behaviors in patients, it helps patients to identify their negative attitudes and obtain new skills to change behaviors, communicate with others, solve problems, change wrong beliefs and attitudes, and reconstruct cognitions [35, 76].

On the other hand, in cognitive behavioral therapy, more desired results are achieved due to the use of such techniques so that the problem-solving technique and muscle relaxation cause self-awareness, and recognition of the psychological aspects of anxiety and stress, leading to reduced symptoms (anxiety and stress). The person’s awareness and knowledge about the effect of negative emotions on mental health, and the advantages of a happy life will cause an increase in performing the tasks learned in therapy sessions, and as a result, depression will be reduced [35]. In general, the effectiveness of CBT in improving the mental health of infertile women can be attributed to the correct thinking way and wrong beliefs, reduced tension, and psychological support of the group. Reduced tension is the result of correcting and adjusting incompatible beliefs and values, cognitive errors, and defective schemas about infertility, and since most infertile patients feel lonely and consider their problems as unique and they cannot talk about their problems, so CBT group sessions are the safest place for emotional discharge and overcoming the feeling of loneliness [77]. Therefore, it is suggested to use CBT preferably in a group as a supplement to infertility medical treatment. According to findings, after CBT, mind-body interventions (with 6 related articles) were used to improve mental health in women with infertility and showed great effects in improving mental health, stress, depression, and anxiety. In explaining the result, it can be said that the interventions based on mindfulness belong to third-generation cognitive behavioral therapies with roots in Eastern religious traditions, especially Buddhism [78]. This style promotes psychological improvement through compromise and flexibility methods and includes interventions that focus on the relationship between the brain, mind, body, behavior, and their effects on health and diseases, and helps the person to be aware of thoughts, feelings, and physical states from moment to moment [79]. Conscious living helps the person to gain a deeper understanding of the realities of life and perceive things “as they are” without attributing expectations, judgments, pessimism, or apprehensions to them, and to see the sufferings, desires, dependence, and instabilities of life [80]. Another prominent feature of this skill is that the person is trained in loving kindness, and the person repeats it during breathing, so it helps to understand experiences in a compassionate and open-minded way [80]. In general, the effectiveness of MBI in improving the mental health of infertile women can be considered to be the result of increasing awareness, paying attention to the body and its movements, and stretching the body to achieve relaxation and mind-body balance; therefore, since it focuses on both mental and physical dimensions, not only it is beneficial for patients with psychological disorders but also to improve wellbeing in normal people [81]. Since personal well-being is the result of balance and harmony of the inner aspects of mind-body-spirit and MBI is a holistic approach, it helps women with infertility experience negative states in new ways and reduces mental distress, and improves mental health [42]. In this regard, it is suggested to use MBI along with infertility medical treatment. According to findings, after CBT and MBI, stress management skills (with 5 related articles) were among the interventions used to promote mental health in women with infertility, and the results indicated the effectiveness of this intervention in improving dimensions of mental health. It can be explained that stress is the body’s uncertain response to any demand and is one of the psychological factors of infertility [6, 7, 82]. Stress management is an individual’s ability to reduce stress and adjust to stressful situations [83]. Stress management interventions contain several interventions including knowledge about stress, identifying inefficient thoughts, self-expression skills, anger, and time management. The purpose of the intervention is to create and develop a set of skills to reduce stress and better deal with the needs and challenges of life [45].

The most important feature of stress management skills (SMS) is helping people to identify the source of stress, precisely evaluate the situations, feelings, and thoughts, and use efficient and problem-solving-based coping styles; as a result, it leads to finding the best solution and provides psychological satisfaction [44]. Since infertile women use emotion-focused coping more to deal with the mental pressure of infertility and have less general health, focusing on stress management skills to make more use of efficient and problem-solving-based coping styles can be effective in improving their mental health [47]. Given that individuals who receive stress management skills can overcome distressing thoughts and experience lower levels of stress, anxiety, and depression, stress management skills (SMS) can be an effective intervention, and it is suggested to use it along with infertility treatment methods.

The search and examination of a variety of psychological interventions in infertility was one of the strengths of the present study. In addition, the research team used two approaches to precisely examine articles in terms of types and contents of interventions, and could identify the most frequent psychological interventions in infertility; however, since it was not possible to access all international databases, a limited number of articles may not have been retrieved, which can be considered as a limitation of this study. Another limitation is that to find relevant articles, articles with an infertile male target group were excluded and only the most common psychological interventions were identified in infertile women, so it is suggested to identify common psychological interventions that promote mental health in infertile men.

Since infertile women face different mental-social challenges and a lack of appropriate interventions and support during treatment influence all aspects of their lives [84], the findings of this research will help specialists and policy-makers in this field to include scientific evidence-based psychological intervention in the care program of infertile women. These can also be used as the foundation for designing training, treatment, counseling, and support programs to reduce psychological disorders and increase adjustment in women with psychological problems caused by infertility.

Given that in most studies, the effectiveness of interventions was evaluated immediately or shortly (1 to 3 months) after finishing the intervention, it is suggested to conduct studies to evaluate the long-term effectiveness of these methods (CBT, MBI, and stress management skills) in infertile women’s psychological empowerment.

## Conclusion

The review of findings of 60 articles not only indicated high diversity of psychological interventions in women with infertility in terms of types and contents of interventions, but also helped to categorize the interventions into 4 categories of CBT, MBI, SMS, and other interventions. According to findings, cognitive behavioral therapy (CBT), mind-body interventions (MBI) and stress management skills (SMS) have been the most frequent and common psychological interventions in infertile women. The application of the most common scientific evidence-based psychological interventions is of great importance to psychologically empower infertile women, especially in societies where women are blamed for infertility, and this causes them to suffer psychological pressure. Due to the importance of having children in line with the population policies of the country and the increasing prevalence of infertility in some societies, including Iran, the use of scientific evidence-based intervention in infertility treatment centers by a team skilled in the field of psychological infertility problems, including reproductive health specialists, psychologists, and midwives is necessary to reduce mental disorders, increase adjustment, and improve the results of infertility treatment. Also, a meta-analysis study on different types of psychological interventions in different aspects of mental health in infertile women is suggested.

### Electronic supplementary material

Below is the link to the electronic supplementary material.

Supplementary Material 1

## Data Availability

The datasets used and/or analyzed during the current study are available from the corresponding author on reasonable request.

## List if Abbreviation

PP: Positive psychology
IVF: In vitro fertilization
IPT: Iranian positive therapy
MBI: Mind-body interventions
SMS: Stress management skills
CBT: Cognitive behavioral therapy
WHO: World Health Organization
EFT: Emotionally focused therapy
PSS: Problemsolving skill training
EWI: Expressive writing intervention
ACT: Acceptance commitment therapy
HPI: Holistic psychological interventions
EPHPP: Effective Public Health Practice Project
PRISMA: Preferred reporting items for systematic reviews and meta-analyses
